# Simple eye-movement feedback during visual search is not helpful

**DOI:** 10.1186/s41235-017-0082-3

**Published:** 2017-11-22

**Authors:** Trafton Drew, Lauren H. Williams

**Affiliations:** Department of Psychology, University of Utah, 380 S 1530 E Beh S 1003, Salt Lake City, UT 84112 USA

**Keywords:** Visual search, Visual attention, Feedback, Eye-tracking

## Abstract

Searching for targets in the visual world, or visual search, is something we all do every day. We frequently make ‘false-negative’ errors, wherein we erroneously conclude a target was absent when one was, in fact, present. These sorts of errors can have tremendous costs, as when signs of cancers are missed in diagnostic radiology. Prior research has characterized the cause of many of these errors as being due to failure to completely search the area where targets may be present; indeed, roughly one-third of chest nodules missed in lung cancer screening are never fixated (Drew, Võ, Olwal, Jacobson, Seltzer and Wolfe, Journal of Vision 13:3, 2013). This suggests that observers do not have a good representation of what areas have and have not been searched prior to declaring an area target free. Therefore, in six experiments, we sought to examine the utility of reducing the uncertainty with respect to what areas had been examined via online eye-tracking feedback. We hypothesized that providing information about what areas had or had not been examined would lead to lower rates of false negatives or more efficient search, namely faster response times with no cost on target detection accuracy. Neither of these predictions held true. Over six experiments, online eye-tracking feedback did not yield any reliable performance benefits.

## Significance

Modern eye-trackers are capable of precisely quantifying where and for how long an observer has looked in a scene. Based on many years of research examining the causes of errors during real-world visual search tasks like diagnostic radiology and baggage screening, there are reasons to believe this information could be very valuable to the searcher. If this promise is met, online eye-tracking feedback could lead to markedly improved visual search performance. This would be of great use in a number of applied venues such as diagnostic radiology and threat detection during military surveillance. However, over six experiments using a variety of different methods of conveying eye-movement information, target prevalence and type of search scene, we obtained no reliable evidence that simple eye-tracking feedback led to any reliable behavioral benefits. In sum, while theory suggested that eye-tracking information could be of use to an observer in a visual search task, we found that simple methods of conveying this information led to no benefit for the observer.

## Background

Visual search is a task that occurs in situations ranging from the mundane (‘search for the pen on your desk’) to the profound (‘search for the sniper’). Costly false negative errors (‘no sniper: we are safe’) occur frequently across different domains. For example, false negatives are a serious problem in screening radiology tasks, where rates of retrospectively visible false negative errors reach 30% in some subspecialties (Wallis, Walsh, & Lee, 1991). What is the cause of these errors? A wealth of visual search research suggests that memory for what areas have and have not been searched is poor. For example, observers often fixate the same items repeatedly before finding a simple target (Gilchrist & Harvey, 2000). In fact, some argue that there is effectively no memory for which items have been rejected as targets during a visual search task (Horowitz & Wolfe, 1998). Although the claim that “*visual search has no memory*” is certainly too strong (Kristjansson, 2000; Peterson, Kramer, Wang, Irwin, & McCarley, 2001; Shore & Klein, 2000), and some argue that the task employed by Horowitz and Wolfe (1998) is flawed (Klein & Dukewich, 2006), it is clear that memory for which areas have been searched is much worse that one might imagine or hope. In fact, when observers were asked to report the locations of their fixations after a 3-second examination of a scene, they were no better at marking the locations of their fixations than they were in guessing the locations of another observer’s fixations (Võ, Aizenman, & Wolfe, 2016).

Given that humans seem to have a poor memory for where they have looked during difficult detection tasks, it seems clear, in principle, that technology could improve performance. Eye-tracking and automated object-detection algorithms are progressing to the point that professional searchers of the future may be wearing glasses that can tell them how recently – if ever – they have examined specific parts of a scene. This possibility leads to two important questions, namely, is this information useful to the searcher? If it is useful, what is the most effective way to convey this information to the searcher?

More information is not always better. At present, it is not clear how to most effectively convey prior search history information to the searcher. Designers of computer-aided detection (CAD) in radiology face an analogous problem. Based on image statistics, each location in a medical image can be assigned a probability of containing an abnormality such as a malignant tumor. At present, CAD systems typically mark areas that exceed a threshold with an arrow or a circle marking the suspicious area (Doi, 2007). Although the CAD systems are good at detecting cancer (almost as good as radiologists), there is a great deal of controversy over whether the use of CAD in clinical settings reliably improves performance (e.g., Cole et al., 2014; Philpotts, 2009). We believe that part of the disappointing performance of the CAD-radiologist system is due to how the information is conveyed to radiologists. Eye-tracking data from our laboratory suggests that the CAD marks attract attention away from areas that were not marked (Drew, Cunningham, & Wolfe, 2012). As a result, performance for targets that occur outside the areas marked by CAD is quite poor; an example of what is called ‘automation bias’ in the human factors literature (Parasuraman & Manzey, 2010).

The current study examined a variety of different methods of conveying eye-movement information back to the user in an effort to improve performance. Although it is certainly not an exhaustive list of all possible methods of conveying this information to the user, this series of studies represents an important first step in determining whether providing this information is useful. Over the course of six experiments, our data surprisingly suggest that simple online feedback during visual search is not helpful. We hope that the demonstration of this lack of a benefit will inspire future researchers to consider alternative methods of conveying information to the user.

## Methods

Each experiment followed a similar design, wherein observers (n = 109 in total) were asked to detect a faint target that was embedded in a search display as quickly and accurately as possible. Observers initiated each trial with a button press. After a short random interval (250–500 ms), the search array was displayed and remained on the screen until the observer either clicked on a location in the search display or a ‘no-target’ rectangle to the left of the search display. Unless otherwise noted, a single target appeared on 25% of trials. Target location was randomized within a 6 by 4 grid (250 × 250 pixel cells) with 50 pixels of random jitter to avoid any sort of predictability for target locations.

Experiments were programmed in MATLAB (version 8.6) Psychtoolbox (version 3.0.12; Kleiner, Brainard, Pelli, & Ingling, 2007). Eye-tracking was performed using an Eyelink 1000 plus recording at 1000 Hz temporal resolution. Raw eye-tracking data was categorized into fixations and saccades using DataViewer Software. Stimuli were presented on a 20’ ASUS flat-screen monitor. Observers carried out the experiment while stabilized by a chin rest 66 cm from the screen. Nine-point calibrations were carried out at the beginning of each block of the experiment and any time the experimenter detected that calibration accuracy was decreasing. Observers who were unable to calibrate to within 0.5 DVA were not allowed to proceed into experimental trials. Those observers that did not complete each block of the experiment were excluded from subsequent analyses. This led to a rejection of a total of 8 out of 117 (6.8%) observers.

In all experiments, the target was a small oval or rectangle (1.25 × 0.66 DVA) that was placed behind a 1500 × 1000 pixel image with 87.5% opacity centered in the screen. The target randomly varied between red or blue, oval or rectangle, and vertical or horizontal orientation. In Experiments 1 and 3–6, images were outdoor scenes. We selected dense outdoor images with few areas of low variability (such as blue skies or calm lakes) because the target was simple to detect in these sorts of areas. Images in Experiment 2 were ‘Clumpy Lumpy Background’ synthetic textures that were designed to emulate real mammogram textures (Castella, Kinkel, Descombes, & Eckstein, 2008). Feedback condition varied across blocks, but within observers in all experiments. Block order was randomized across observers.

**Fig. 1:**
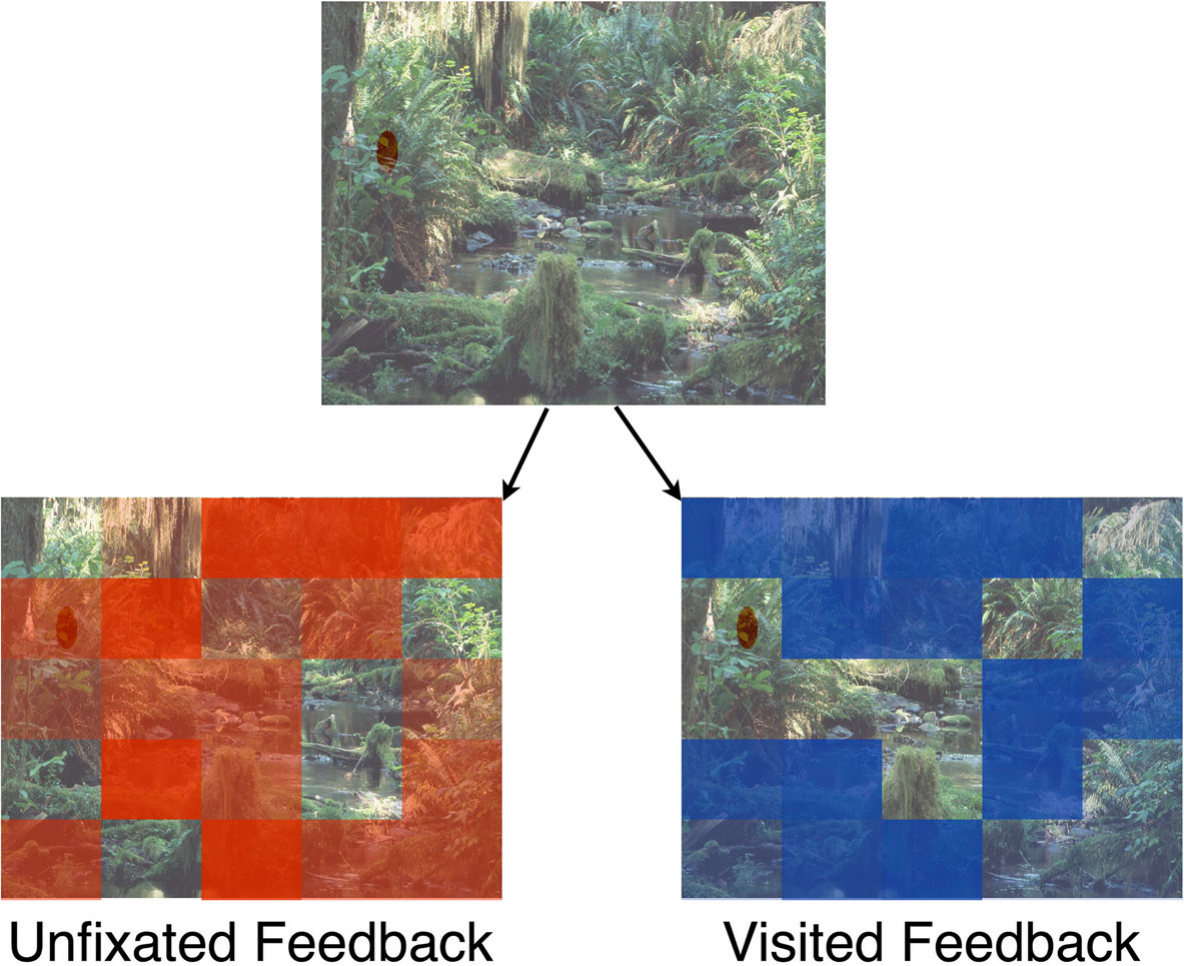
Schematic representation of feedback systems employed in Experiments 1–3. The red target has been made larger and easier to detect for display purposes

In Experiments 1–3, we examined two types of feedback – ‘Unfixated’ and ‘Visited’. Feedback condition varied across blocks, the order of which was randomized across observers. In Experiments 1–3, feedback could be toggled ‘on’ by holding the tab button down. Otherwise, the feedback was not visible. Thus, feedback on each trial began ‘off’ until the observer chose to turn the feedback ‘on’ by holding the tab button down. In both conditions, observers were instructed that errors in these tasks are often the result of a target that was never fixated, and that these feedback systems were designed to help decrease these sorts of errors. By manipulating the opacity of a 6 by 4 grid overlaid on the search array, Unfixated feedback indicated to the observer which areas of the screen had not been fixated. Thus, the opacity of the red feedback mask for an area that had been fixated for 0 ms was 39%. As result, the screen would be a uniform translucent red if the feedback was deployed at the beginning of the trial. Opacity of the feedback mask then decreased as the cumulative dwell time in that region increased until it reached 0%. The Visited feedback block followed the same scheme in reverse, wherein regions that were not visited had a feedback mask opacity of 0%. This number then increased to a maximum of 39% as dwell time in this region increased. Therefore, in Fig. 1, the visited feedback indicates to the user that the area where the target is located has not been fixated.

Whereas online feedback was only provided when the observer pressed the ‘tab’ button in Experiments 1–3, in Experiments 4–6 the feedback was automatically provided to the user in one of two manners. In Experiment 4, the entire search array was initially masked by semi-opaque grey rectangles. As the observer gazed in these regions, the opacity in each area decreased from 84% to 0%. In Experiments 5 and 6, once the observer had recorded a response for a trial, they were shown an additional screen highlighting the 10 grid regions that they had visited for the least amount of time. All other areas were rendered invisible in order to encourage the observer to evaluate the areas that had previously been evaluated for the least amount of time. They were then allowed to amend their initial response.

The progression from Experiment 1–6 is outlined in Table 1. Over the course of these experiments, we varied target prevalence, image type, and method by which feedback was conveyed to the observer. We will return to the differences across experiments in the Discussion section.

**Table 1:** Experiment overview

Experiment	Number of observers	Target prevalence	Images used	Feedback type	Trials per block
1	15	25%	Real scenes	Unfixated, visited	48
2	14	25%	1/f noise	Unfixated, visited	48
3	19	50%	Real scenes	Unfixated, visited	48
4	16	25%	Real scenes	Fade	48
5	23	25%	Real scenes	Visited reminder	60
6	22	50%	Real scenes	Visited reminder	60

## Results

The primary results are highlighted in Table 2. In order to assess whether eye-tracking feedback led to a reliable benefit in finding targets, we computed a composite measure of corrected accuracy by subtracting False Alarm Rate from Hit Rate. The pattern of results is identical using d-prime as the primary outcome measure. Eye-tracking feedback was associated with a reliable benefit in only one of the six experiments. The feedback system in Experiments 5 and 6 was designed to highlight areas that were not examined prior to making a decision so that the Observer could amend their response after receiving an additional opportunity to review these regions. We were therefore surprised to find that Hit Rate on target-present trials for which the target region was highlighted was worse (46%) than when no feedback was given (71%, t(22) = 1.62, *P* = 0.11).

**Table 2:** Behavioral performance and associated statistics

Experiment	Hit rate (HR)			False Alarm (FA) rate			Accuracy (HR-FA)			F value	*P* value	Bayes factor evidence for H_0_
	Unfixated feedback	No feedback	Visited feedback	Unfixated feedback	No feedback	Visited feedback	Unfixated feedback	No feedback	Visited feedback			
1	0.53	0.55	0.49	0.08	0.09	0.08	0.45	0.46	0.42	0.39	0.68	5.38
2	0.91	0.90	0.90	0.90	0.10	0.10	0.90	0.88	0.88	0.37	0.70	4.62
3	0.78	0.77	0.76	0.03	0.04	0.03	0.76	0.73	0.73	0.42	0.66	5.38
4	n/a	0.58	0.56	n/a	0.05	0.05	n/a	0.51	0.52	0.15	0.70	5.07
5	0.71	0.80	n/a	0.05	0.02	n/a	0.79	0.66	n/a	10.21	0.004*	0.11
6	0.87	0.86	n/a	0.03	0.02	n/a	0.84	0.85	n/a	0.04	0.84	6.12

*s indicate statisitcally signficant *p*-values

As a result of this unexpected finding and the difference between the results from Experiment 5 and the prior experiments, we attempted to replicate and extend this finding at higher target prevalence (increasing from 25% to 50%). As in four of the previous five examinations of this effect, Experiment 6 yielded no apparent benefit of eye-tracking feedback. We will return to the interpretation of Experiment 5 in the Discussion section below.

It is difficult to interpret null-results using traditional null-hypothesis testing approaches (Wagenmakers, 2007). In order to provide more information with respect to whether our hypothesis that feedback would lead to improved performance compared to the No Feedback condition, we computed Bayes Factors (BF) using the Jeffery–Zellner–Siow prior to evaluate whether the evidence favored the null hypothesis (feedback type makes no difference to performance) or the ‘full’ model (Rouder, Morey, & Speckman, 2012). BF for experiments 1–3 and 6 ranged from 4.62 to 6.12, providing substantial evidence against the ‘full’ model. Experiment 5 was associated with a BF of 0.011 (alternatively a BF of 9.35 in favor of our H_1_, where feedback reliably alters performance). This is typically categorized as ‘moderate evidence’ in favor of the ‘full’ model (Wetzels & Wagenmakers, 2012).

In order to assess whether the eye-tracking feedback was utilized, we analyzed Response Time (RT) on correct trials in Experiments 1–3 in a series of 3 (Feedback Type) × 2 (Target Number) repeated measure ANOVAs. Prior to analyzing this data, we filtered out RTs that were < 200 ms and > 60 s. Given the positive skew of RT distributions, we did not exclude RTs based on standard deviations of the RT distribution (Palmer, Horowitz, Torralba, & Wolfe, 2011). The goal of our filtering was to remove aberrant trials where the observer either inadvertently responded too quickly, or became distracted during the trial (Wolfe, Boettcher, Josephs, Cunningham, & Drew, 2015). These results are summarized in Table 3. Overall, while target-absent trials were reliably longer than present trials (all F > 35, all *P* < 0.001), there was no effect of Feedback Type (all F < 2.4, all *P* > 0.11). Ideally, eye-tracking feedback would increase accuracy while not increasing RT. In the absence of a reliable accuracy benefit, the lack of an RT effect is more difficult to interpret. One concern may be that there was no increase in RT because the observers were not using the feedback. Although there was substantial variability in terms of how often and for how long the feedback was turned ‘on’ via the ‘tab’ button, our results are qualitatively identical (no benefit of feedback) if we focus on those observers who used the feedback frequently.

**Table 3:** Response time statistics

Experiment	Target presence	Feedback type	Interaction
1	F(1,14) = 35.7, *P* < 0.001	F(2,28) = 1.54, *P* = 0.23	F(2,28) = 0.69, *P* = 0.51
2	F(1,18) = 79.7, *P* < 0.001	F(2,36) = 0.7, *P* = 0.499	F(1,15) = 55.0, *P* < 0.001
3	F(1,12) = 45.2, *P* < 0.001	F(2,24) = 2.39, *P* = 0.11	F(2,24) = 3.43, *P* = 0.05
4	F(1,15) = 214.4, *P* < 0.001	F(1,15) = 55.0, *P* < 0.001	n/a

**Fig. 2:**
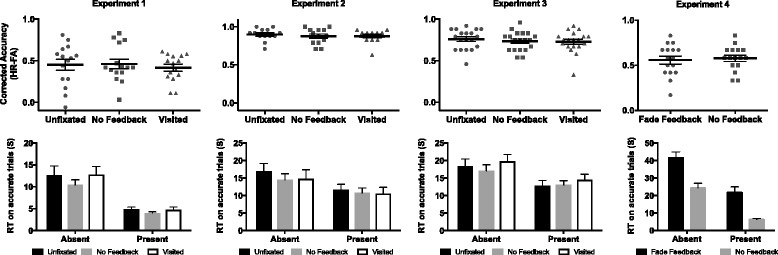
Results for Experiments 1–4. Error bars represent standard error of the mean. Note the varying scales on the response time graphs

**Fig. 3:**
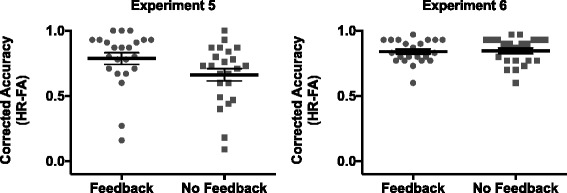
Results for Experiments 5 and 6. Error bars represent standard error of the mean

Experiment 4 was conducted to address the concern that not all observers were using the feedback as intended. In this experiment, feedback was compulsory – the Unfixated feedback system from Experiments 1–3 was always engaged such that observers had to fixate on an area in order to reduce the opacity of what was effectively a translucent mask in front of the search stimulus. As expected, this version of feedback led to a large increase in RT (F(1,15) = 54.95, *P* < 0.001) but, as previously outlined, no benefit on accuracy (F(1,15) = 0.15, *P* = 0.7). In sum, over the course of Experiments 1–4, we did not observe any evidence that online feedback led to more efficient search by reducing the amount of time wasted evaluating previously evaluated regions (Fig. 2). This is consistent with previous work by Dickinson & Zelinsky (2005), who mostly focused on distractor refixations to reach essentially the same conclusion. We did not analyze RT in Experiments 5 and 6 because the feedback system in both experiments naturally led to a second viewing epoch, thereby rendering comparisons of overall time spent less interesting (Fig. 3).

## Discussion

There is a significant societal cost associated with missed targets in fields as varied as radiology (Berlin, 1996), baggage screening (Wolfe, Brunelli, Rubinstein, & Horowitz, 2013), and military surveillance (Shanker & Richtel, 2011). Given the increasing popularity and decreasing costs of eye-tracking technology (Duchowski, 2017), there is great potential for this relatively simple technology to help reduce the rate of miss errors. Moreover, there is converging evidence from the cognitive psychology literature that observers have poor memory for where they have searched (Horowitz & Wolfe, 1998; Võ et al., 2016), and many errors are caused by simply never looking at the target (Kundel, Nodine, & Carmody, 1978; Rich et al., 2008). It therefore stands to reason that providing detailed information with respect to where an observer has looked would lead to substantial benefits in terms of decreased miss rates, or decreased time spent examining images thanks to limiting the number of repeated fixations on areas that have already been examined. Across six experiments, we found essentially no evidence for either of these predictions.

The lone exception was Experiment 5, where feedback about what areas of the image had not been searched was provided to the observer after they had made an initial response. Although there was a significant benefit in this experiment, there was no hint of a benefit in Experiment 6, which was an exact replication except that the target prevalence was increased from 25% (Experiment 5) to 50% (Experiment 6). It is notable that Peltier and Becker (in press) found no benefit in three of the four studies where they also examined the utility of eye-movement feedback during low prevalence visual search. Along similar lines, Experiments 1, 3, and 4 were also conducted at 25% prevalence and there was no benefit of feedback in any of those experiments. Finally, if the feedback provided in Experiment 5 was helpful, we expected the benefit to be due to the feedback alerting the observer they had not fixated on the area that contained the target. Our data indicate that this was not the case on most trials. In fact, the target location was highlighted by our feedback system on only approximately 8% of all target-present trials. Performance on those few trials where the target position was highlighted as having not been properly evaluated was no better than on trials where the target was not highlighted. We therefore conclude that the most likely interpretation of this experiment is that it is a false positive.

Our data suggest that, across a variety of simple manipulations, eye-tracking feedback does not appear to be useful during a difficult target detection task. There are a number of reasons why our attempts to provide evidence that eye-tracking feedback is useful were not successful. Many of these potential reasons are due to design decisions we made in an attempt to demonstrate the utility of this approach on a paradigm that could reasonably be expected to scale up to more realistic scenarios, such as searching a chest radiograph for signs of cancer, or surveying a pathway in search of evidence of improvised explosive devices.

Five of the six experiments were conducted with real-world outdoor scenes. This naturally meant that some areas were more salient than others, and targets were easier to detect in some regions than others. Thus, the feedback we provided had to compete with the natural inclination to search certain regions based on salience and other considerations. Experiment 2 was aimed at addressing this concern by employing a variant of 1/f noise with none of the additional information that accompanies real world scenes, i.e. scene structure. The results of Experiment 2 show that, even when the search array was simple 1/f noise with no semantic information, our feedback scheme was not effective.

Search slope, or efficiency, is a common method to evaluate visual search performance. Search efficiency is typically defined as the slope between the number of distractors in an array and RT. In the current work, we were not able to evaluate this metric because we used real scenes and 1/f noise scenes that did not contain a discrete number of distractors (though, of course, real scenes contain a great deal of less clearly defined distractors (Russell, Torralba, Murphy, & Freeman, 2008)). We were not interested in the efficiency of search but the overall accuracy and speed with which the search was conducted. Memory of which distractors have already been examined could provide an avenue for eye-tracking feedback to accelerate search performance. Previous work tested this idea in a series of experiments where fixated distractors were deleted from the scene (Dickinson & Zelinsky, 2005). The authors reasoned that, if search is memoryless, then eliminating fixated distractors should reduce unnecessary re-fixations and improve search efficiency. However, similar to the current study, they found that their intervention yielded no benefit relative to control conditions with no eye-tracking feedback.

### Future directions

When targets were present in the current work, the location was random within the scene and feedback was blind to the location of the target. One would certainly expect that, if the feedback system was aware of the target location and provided feedback with respect to whether or not that area had been fixated, it would lead to better outcomes. However, there does not appear to be any simple way to scale such a system up to a real world situation where target locations are unknown. Recent advances in computer vision algorithms present one potential pathway for providing observers with ‘smart’ feedback that parses scenes into discrete areas and modifies feedback relative to the likelihood that a specific threat may occur in that area. For instance, an improvised explosive device is unlikely to be placed in a pond. Perhaps a system that combined well-researched priors about likely target locations with ongoing eye-movements would yield a system that improved overall search efficiency.

A limitation of the current approach is that it assumes that, if an observer examines a target, they will detect it. Clearly, this is not always the case. Observers often fixate on the unexpected stimulus, which is missed in the inattentional blindness literature (e.g. Drew et al., 2013). Fixated targets are often not detected during low prevalence visual search tasks (Hout, Walenchok, Goldinger, & Wolfe, 2015). Foundational work in the medical image perception literature by Kundel et al. (1978) categorized target miss errors as caused by “*search, recognition or decision*” errors. The current approach was designed to reduce ‘search’ errors, which occur when the observer never fixates the target. However, this approach would not be helpful for recognition or decision errors, where the target is fixated but not identified. The ratio of search errors to recognition and decision errors varies across search task. In the current work, we adopted a search task with complex, real-world stimuli and simple targets that did not vary much across trials. It is possible that our eye-movement feedback protocols would have been more useful in a search task specifically chosen to elicit a high proportion of ‘search’ errors.

Although eye-movement feedback does not appear capable of improving search in the current task through highlighting what areas have or have not been searched, it may hold promise in other implementations. For instance, one of the most reliable factors to differentiate good airport baggage screeners from poor ones is RT variability (Biggs, Cain, Clark, Darling, & Mitroff, 2013). This may be why systematic search is generally less vulnerable to miss errors (Mitroff, Biggs, & Cain, 2015). Eye-movement feedback could be relatively simply adapted to provide information about the systematicity of search. Along similar lines, recent work has suggested that using eye-movement data to inform the searcher when to quit may lead to improvements in search efficiency (e.g., Deza, Peters, Taylor, Surana, & Eckstein, 2017). Future research will be necessary to determine if either of these approaches leads to a reliable and generalizable benefit to search performance.

## Conclusion

While our data convincingly demonstrate that simple eye-movement feedback was not helpful in aiding the detection of targets in a difficult visual search task, it would be premature to conclude that eye-tracking offers no promise for improving performance in these sorts of tasks. A wealth of prior evidence from the visual search literature suggests that information about where one has looked should be valuable to the observer. The central challenge of this problem may lie in uncovering the optimal methods for conveying this information to the user.
